# Eritoran Attenuates Hepatic Inflammation and Fibrosis in Mice with Chronic Liver Injury

**DOI:** 10.3390/cells10061562

**Published:** 2021-06-21

**Authors:** Yun-Cheng Hsieh, Kuei-Chuan Lee, Pei-Shan Wu, Teh-Ia Huo, Yi-Hsiang Huang, Ming-Chih Hou, Han-Chieh Lin

**Affiliations:** 1 Division of Gastroenterology and Hepatology, Department of Medicine, Taipei Veterans General Hospital, Taipei 11217, Taiwan; ychsieh7@vghtpe.gov.tw (Y.-C.H.); pswu6@vghtpe.gov.tw (P.-S.W.); tihuo@vghtpe.gov.tw (T.-I.H.); yhhuang@vghtpe.gov.tw (Y.-H.H.); mchou@vghtpe.gov.tw (M.-C.H.); hclin@vghtpe.gov.tw (H.-C.L.); 2 Department of Medicine, School of Medicine, National Yang Ming Chiao Tung University, Taipei 11217, Taiwan; 3 Institute of Pharmacology, School of Medicine, National Yang Ming Chiao Tung University, Taipei 11217, Taiwan

**Keywords:** toll-like receptor 4, receptor antagonist, hepatic stellate cell, chronic liver disease, animal model

## Abstract

Toll-like receptor 4 (TLR4) signaling plays a key role in liver inflammation and fibrosis. The therapeutic effects of eritoran, a TLR4 antagonist, in mice with chronic liver injury remained unclear. C57BL/6 mice were fed a fast-food diet (FFD) or treated with carbon tetrachloride (CCl_4_) to induce chronic liver injury. Eritoran (10 mg/kg) or a vehicle was randomly intraperitoneally administered to the FFD-fed mice and the CCl_4_-injured mice. Primary mouse liver cells were cultured with lipopolysaccharide (LPS) or eritoran. In both FFD and CCl_4_ mouse models, eritoran significantly reduced serum ALT levels and decreased hepatic inflammatory cell infiltration without altering hepatic steatosis. Additionally, eritoran attenuated liver fibrosis by decreasing hepatic stellate cells (HSCs) activation and the abundance of α-smooth muscle actin and transforming growth factor-β1. Hepatic TLR4 downstream signaling including MyD88 expression, NF-κB p65 nuclear translocation, p38 and JNK phosphorylation were successfully inhibited by eritoran. In the in vitro study, LPS-induced nuclear translocation of NF-κB in primary HSCs and Kupffer cells was significantly suppressed by eritoran. In conclusion, eritoran attenuated hepatic inflammation and fibrosis by inhibition of the TLR4 signaling pathway in mice with chronic liver injury. Eritoran may serve as a potential drug for chronic liver disease.

## 1. Introduction

Chronic liver disease (CLD) is a continuous process of inflammation and the healing process of liver parenchyma which leads to fibrosis and cirrhosis, imposing a high morbidity and mortality burden worldwide [1]. In addition to the treatment of the underlying liver disease, new drugs have been developed to target different steps in the pathophysiology of chronic liver injury, such as liver metabolism, apoptosis, inflammation and fibrosis [2]. Nevertheless, only a minority of patients achieve treatment response [2]. Therefore, novel effective drugs for CLD are needed.

Toll-like receptor 4 (TLR4) is a pattern recognition receptor that activates the innate immune system by recognizing its major ligand, lipopolysaccharide (LPS) [3]. In the liver, TLR4 is expressed in parenchymal and non-parenchymal cells [4]. CLD, such as nonalcoholic steatohepatitis (NASH), is highly associated with the triggering of TLR4 signaling by gut-derived bacteria and cell death products [5,6,7]. Upon ligand binding, TLR4 recruits downstream adaptor molecules, including myeloid differentiation factor 88 (MyD88) and toll/interleukin-1 receptor domain-containing adaptor-inducing interferon-β [5]. MyD88-dependent signaling results in NF-κB activation, mitogen-activated protein kinase cascades and the related inflammatory cytokine production, whereas MyD88-independent signaling regulates interferon regulatory factor leading to cytokine and interferon secretion [8,9]. In animal models of chronic liver injury, suppression of the TLR4 signaling pathway via modulation of LPS production, TLR4 expression or downstream signaling molecules has been shown to ameliorate liver injury [5]. In in vitro studies, Kupffer cells (KCs) express TLR4 and respond to LPS by releasing proinflammatory and profibrogenic mediators and reactive oxygen species [10]. TLR4 activation in hepatic stellate cells (HSCs) upregulates chemokine production and induces the chemotaxis of KCs [11]. LPS also sensitizes HSCs to transforming growth factor-β (TGF-β) signals, which mediate fibrogenesis through the TLR4–MyD88–NF-κB pathway [11]. This evidence suggests that TLR4 signaling plays an important role in hepatic inflammation and fibrosis and promotes the progression of CLD [12].

Eritoran tetrasodium (E5564), a TLR4 receptor antagonist, is a synthetic lipid A analog of *Rhodobacter sphaeroides* that competes with LPS for binding to the hydrophobic pocket of the MD2 portion of the TLR4 receptor complex [13]. It has been shown that the binding of eritoran to the TLR4/MD2 complex blocks the activation of NF-κB and the production of proinflammatory cytokines, such as TNF-α and interleukin (IL)-6, both in vivo and in vitro, in response to LPS [14,15,16,17,18]. Eritoran has been found to block TLR4-mediated inflammation in acute liver failure [19] and liver ischemia/reperfusion injury models [20] and attenuate liver damage in a hemorrhagic/shock model [21]. However, the effect of eritoran on chronic liver injury has not yet been reported. In this study, we examined whether chronic administration of eritoran blocks hepatic TLR4 signaling, the subsequent inflammatory responses and fibrosis in mouse models of chronic liver injury.

## 2. Materials and Methods

### 2.1. Animals

Adult male C57BL/6 mice (National Laboratory Animal Center, Taipei, Taiwan) aged 8–10 weeks were used in all the experiments. The mice were caged at 22 °C with a 12-h light/dark cycle and allowed free access to food. The study was approved by the Animal Experiment Committee of Taipei Veterans General Hospital and performed in accordance with the Guides for the Care and Use of Laboratory Animals prepared by the National Academy of Sciences (Washington, DC, NW, USA).

### 2.2. Study Design

**Figure 1:**
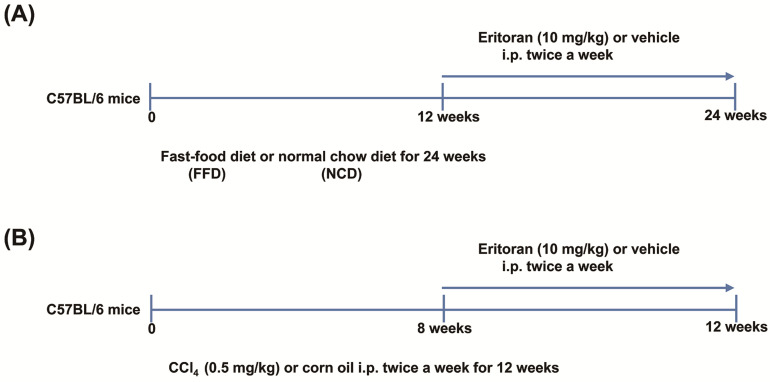
The experimental protocols of animal studies. (**A**) C57BL/6 mice were fed a fast-food diet (FFD) or normal chow diet (NCD) for 24 weeks. After 12 weeks of FFD or NCD feeding, the mice were randomly assigned to receive eritoran (E: 10 mg/kg) or the vehicle (V: 100 μL normal saline) twice a week via intraperitoneal injection for 12 weeks with continuous FFD or NCD feeding (NCD-V: *n* = 6; NCD-E: *n* = 6; FFD-V: *n* = 10; FFD-E: *n* = 9). (**B**) C57BL/6 mice were intraperitoneally administered carbon tetrachloride (CCl_4_) (0.5 mg/kg body weight twice a week) or corn oil (control, Ctrl) for 12 weeks. After eight weeks of CCl_4_ or corn oil treatment, the mice were randomly administered eritoran (E: 10 mg/kg) or the vehicle (V: 100 μL normal saline) intraperitoneally twice a week for four weeks, with continuous CCl_4_ or corn oil treatment (Ctrl-V/Ctrl-E: *n* = 8; CCl_4_-V/CCl_4_-E: *n* = 9).

The mice were fed a fast-food diet (FFD, 20% fat, 49.9% carbohydrate, 17.8% protein, 2% cholesterol and 5% fiber (AIN-76 Western Diet, test diet)), glucose (18.9 g/L) and fructose (23.1 g/L) for 24 weeks to generate NASH and liver fibrosis [22]. Following 12 weeks of FFD or normal chow diet (NCD) feeding, the mice were randomly assigned to receive eritoran (Eisai, Inc., Andover, MA, USA) (10 mg/kg) [20] or the vehicle (saline, 100 μL) twice per week via intraperitoneal injection for 12 weeks with continuous FFD or NCD feeding (Figure 1A).

To validate the effects of eritoran on established liver fibrosis, a carbon tetrachloride (CCl_4_) mouse model was also used. The mice were intraperitoneally administered CCl_4_ (0.5 mg/kg body weight twice a week) or corn oil (served as the control) for eight weeks and then received eritoran (10 mg/kg) or the vehicle (saline, 100 μL) intraperitoneally twice a week for four weeks, with continuous CCl_4_ or corn oil treatment (Figure 1B). At the end of the experiment, all the mice were sacrificed for the analysis.

### 2.3. LX-2 Cell Studies

The effective dose and possible cell toxicity of eritoran in HSCs were tested in a human activated HSC cell line, LX-2 cells (Merck Millipore, Burlington, MA, USA). LX-2 cells were incubated in the Dulbecco’s modified Eagle’s medium (DMEM) with the vehicle, eritoran (10 ng/mL, 100 ng/mL, 1 μg/mL and 10 μg/mL), with or without LPS (100 ng/mL) for 6 and 24 h.

Cell viability was measured using the methyl thiazolyl tetrazolium (MTT) assay (Sigma-Aldrich, Inc., St. Louis, MO, USA). MTT was dissolved in phosphate-buffered saline to a 5 mg/mL concentration. After the addition of 20 μL MTT solution and 2 h of incubation, the medium was aspirated, and the formed MTT crystal violet was dissolved in dimethyl sulfoxide. The MTT metabolite amount was determined using a microplate reader at the absorbance of 570 nm (SpectraMax 250, Molecular Devices, CA, USA).

### 2.4. Primary Cell Isolation and Culture

Primary HSCs and KCs were isolated from the mice treated with CCl_4_ (0.5 mg/kg body weight twice a week) intraperitoneally for eight weeks using collagenase–pronase perfusion and subsequent density centrifugation using Nycodenz gradients as previously described [23,24,25]. The isolated HSCs were seeded on six-well plates (3 × 10^5^ HSCs/well) and cultured in the DMEM containing 10% fetal bovine serum (FBS) and antibiotics at 37 °C in a humidified atmosphere containing 95% air and 5% CO_2_. Twenty-four hours after isolation, HSCs were treated with the vehicle, LPS (100 ng/mL, Sigma-Aldrich, Inc., St. Louis, MO, USA) [11] or LPS plus eritoran (10 μg/mL) for 24 h.

KCs were selected as F4/80-expressing cells using MACS (Miltenyi Biotec) [11]. The procedure led to a 95% purity of KCs, which was confirmed using flow cytometry for F4/80 expression. The isolated KCs were seeded on six-well plates (3 × 10^5^ KCs/well) and cultured in the RPMI-1640 medium containing 10% FBS and antibiotics. Twenty-four hours after seeding, KCs were treated with the vehicle, LPS (100 ng/mL) or LPS plus eritoran (10 μg/mL) for 24 h.

Primary hepatocytes were isolated from the male C57BL/6 mice by modifying a previously described collagenase perfusion method [26]. Twenty-four hours before plating the cells, cell culture plates were coated with 0.1% rat-tail collagen (Sigma-Aldrich). The primary hepatocytes were seeded on six-well plates (2 × 10^5^ cells/well) and cultured in the DMEM containing 10% FBS and antibiotics. Twenty-four hours after seeding, the hepatocytes were treated with the vehicle, LPS (1 μg/mL) [27] or LPS plus eritoran (10 μg/mL) for 24 h.

### 2.5. Measurement of Blood Biochemistry and Lipopolysaccharide

Serum alanine aminotransferase (ALT), glucose and cholesterol levels were measured using a standard auto-SMAC analyzer (Cobas 8000; Roche Diagnostics GmbH, Mannheim, Germany). Insulin levels were determined using a mouse insulin ELISA kit (Crystal Chem Inc., Downers Grove, IL, USA). LPS levels were measured using an ELISA kit (Cloud-Clone Corp, Katy, TX, USA).

### 2.6. Real-Time Quantitative Reverse Transcription Polymerase Chain Reaction (RT-PCR)

Nucleotide sequences of the primers used in this study are shown in Table S1. Gene expression levels were quantitatively measured using an ABI PRISM 7900HT Sequence Detection System (Applied Biosystems Inc. Foster City, CA, USA) using SYBR Green. The specificity of each PCR product was evaluated by the melting curve analysis followed by agarose gel electrophoresis.

### 2.7. Histological Studies

Liver tissues were fixed for 24 h with 10% paraformaldehyde at 22–28 °C, then dehydrated, embedded in paraffin, cut into 4 μm-thick slices and stained with hematoxylin and eosin (H&E).

For immunohistochemical (IHC) staining, the slides were incubated at 4 °C overnight with the primary antibodies shown in Table S2. After overnight incubation, the slides were incubated with the corresponding secondary antibodies for 30 min. The slides were stained with a supersensitive polymer HRP IHC detection system (BioGenex Laboratories Inc., Fremont, CA, USA) and then counterstained with Mayer’s hematoxylin. The images were captured with a microscope (Olympus, AX-80) and the Olympus CellSens imaging software.

### 2.8. Western Blot Analysis

Protein extraction was performed according to the standard methods, and nuclear protein was extracted using NE-PER Nuclear extraction reagents (Thermo Scientific, Waltham, MA, USA) [28]. The blots were incubated with the primary antibodies shown in Table S2. After washing, the membranes were incubated with horseradish peroxidase-conjugated goat anti-rabbit secondary antibodies (Jackson ImmunoResearch Laboratories Inc., West Grove, PA, USA) for the rabbit primary antibodies for 1 h. Subsequently, the blots were developed by enhanced chemiluminescence (ECL Western Blotting Analysis System, Amersham, UK). The intensities of the bands of interest were analyzed using the ImageJ software (National Institutes of Health, Bethesda, MD, USA).

### 2.9. Sirius Red Staining

Sirius Red staining was performed using a Sirius Red Staining Kit (Polysciences Inc., Warrington, PA, USA).

### 2.10. Measurement of Hepatic Hydroxyproline Levels

Hydroxyproline levels were determined using a Hydroxyproline Colorimetric Assay Kit (BioVision Inc., Milpitas, CA, USA). Liver tissues (20 mg) were homogenized and hydrolyzed with 200 μL of 12 N HCl in a pressure-tight Teflon-capped vial at 120 °C for 3 h. After transferring 10 μL of each hydrolyzed sample to a 96-well plate to dry at 65 °C, 100 μL chloramine T reagent was added to each sample, and the plate was incubated at around 22–28 °C for 5 min. After adding 100 μL DMAB reagent to each well and incubating for 90 min at 60 °C, the absorbance of all the samples was measured at 560 nm using a microplate reader.

### 2.11. Measurement of Hepatic Steatosis

Triglyceride content in the livers was measured using a triglyceride colorimetric assay kit (Cayman Chem Inc., Ann Arbor, MI, USA). Oil Red O staining was performed on cryosections (8 μm) incubated in 60% isopropanol for 30 s and in the Oil Red O solution (Muto Pure Chemicals, Co., Ltd., Tokyo, Japan) at 37 °C for 10 min. Specimen integrity was verified by staining with Mayer’s hematoxylin for 2 min.

### 2.12. Glucose Tolerance and Insulin Tolerance Tests

After a 16-h fast, glucose tolerance tests were performed after intraperitoneal injection of D-glucose (Sigma-Aldrich, Inc., St. Louis, MO, USA) at the dose of 2 mg/g body weight. For the insulin tolerance test, the mice were injected with regular human insulin (Santa Cruz Biotechnology, Inc., CA, USA) at the dose of 0.75 U/kg body weight after a 6-h fast. Blood glucose levels were measured with a portable blood glucose meter (OneTouch Ultra 2, LifeScan, Johnson & Johnson, New Brunswick, NJ, USA).

### 2.13. Flow Cytometry

The mouse-specific antibodies used are shown in Table S2. Mouse Fc block (eBioscience, San Diego, CA, USA) was used to block binding of aggregated immunoglobulins to Fc receptors. Flow cytometry was performed using a FACSCanto II cytometer (BD Biosciences, San Jose, CA, USA).

### 2.14. In Vitro Small Interfering RNA (siRNA) Transfection

To silence MyD88 expression in primary isolated KCs, mouse MyD88-siRNA or siRNA control (Santa Cruz Biotechnology Inc., Paso Robles, CA, USA) was used for transfection using Lipofectamine RNAiMAX (Thermo Fisher Scientific, Waltham, MA, USA). Briefly, siRNAs and Lipofectamine RNAiMAX were mixed and incubated at 22–28 °C for 5 min in the Opti-MEM medium (Thermo Fisher Scientific, Waltham, MA, USA). The mixture was then transferred to a six-well plate with KCs (4 × 10^5^/well) at the final concentration of 30 nmol/L (siRNA) and 7.5 μL RNAiMAX per well. After siRNA transfection for 48 h, the cells were incubated with or without LPS (10 ng/mL; Sigma, St. Louis, MO, USA) or eritoran (10 μg/mL) for 6 h and subsequently collected for Western blot analysis.

### 2.15. Statistical Analysis

The data were analyzed using GraphPad Prism 8 (GraphPad Software, San Diego, CA, USA) and are expressed as the means ± SEM. Statistical significance between the groups was determined using the Kruskal–Wallis test followed by Dunn’s tests. Significance was determined at the *p*-value < 0.05.

## 3. Results

### 3.1. Eritoran-Attenuated Hepatic Inflammation and Fibrosis in the FFD-Fed Mice

**Figure 2:**
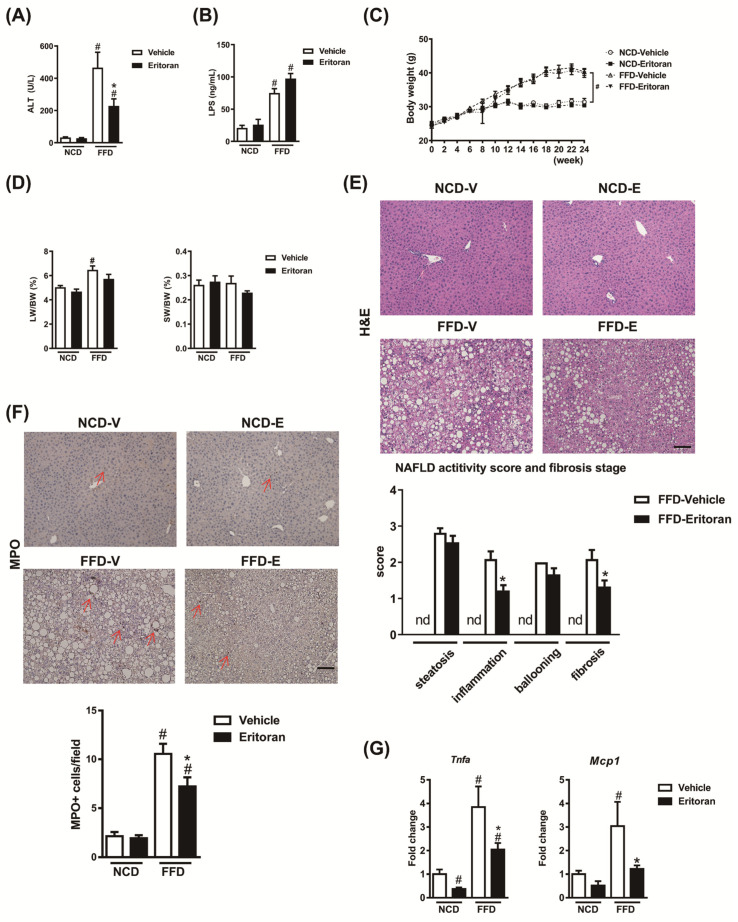
Eritoran reduced hepatic inflammation in the mice fed a fast-food diet. (**A**) Serum alanine aminotransferase (ALT) levels. (**B**) Serum lipopolysaccharide (LPS) levels. (**C**) Body weight (BW), (**D**) liver weight (LW) and spleen weight (SW). (**E**) Hematoxylin and eosin (H&E) and (**F**) myeloperoxidase (MPO) staining of the liver sections and quantification of NAFLD activity scores as well as MPO-positive stained cells (arrow). Scale bar = 100 μm; nd: non-detectable. (**G**) Hepatic transcript levels of tumor necrosis factor-α (*Tnfa*) and monocyte chemoattractant protein 1 (*Mcp1*). NCD-V/NCD-E: the mice fed a normal chow diet (NCD) were injected with a vehicle (NCD-V, *n* = 6) or eritoran (NCD-E, *n* = 6); FFD-V/FFD-E: the mice fed a fast-food diet (FFD) were injected with a vehicle (FFD-V, *n* = 10) or eritoran (FFD-E, *n* = 9); ^#^ *p* < 0.05 vs. NCD-V; * *p* < 0.05 vs. FFD-V.

Compared to the NCD-fed mice, the FFD-fed mice exhibited an increase in the serum ALT and LPS levels (Figure 2A,B). Eritoran significantly decreased serum ALT levels (Figure 2A) without altering serum LPS levels (Figure 2B) in the FFD-fed mice. The kinetic body weight change, liver weight, spleen weight (Figure 2C,D) and intake of the FFD (FFD-V: 2.35 ± 0.05 g/mouse/day vs. FFD-E: 2.3 ± 0.19 g/mouse/day) did not differ between the FFD-V and FFD-E groups. H&E and myeloperoxidase (MPO) staining of the liver sections showed that eritoran treatment significantly reduced lobular inflammation and intrahepatic neutrophil infiltration in the FFD-fed mice (Figure 2E,F). The hepatic transcript levels of *Tnfa* and monocyte chemoattractant protein 1 (*Mcp1*) were decreased in the eritoran-treated FFD-fed mice (Figure 2G).

**Figure 3:**
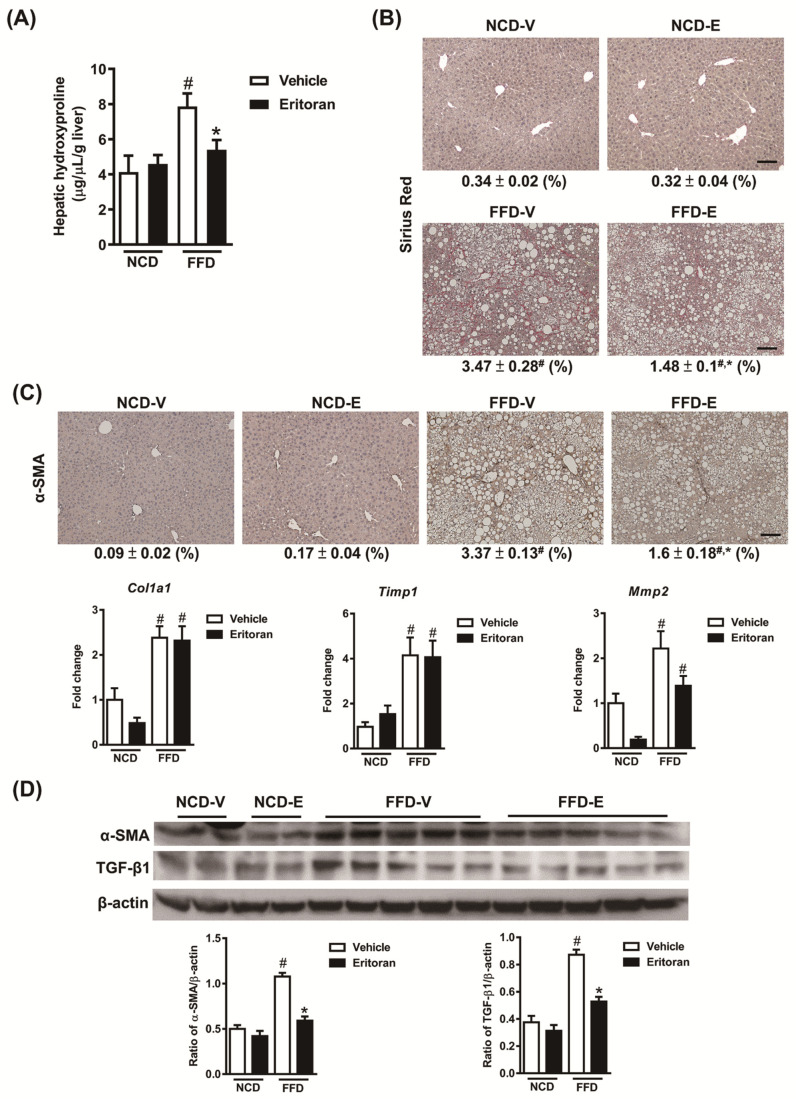
Eritoran attenuated liver fibrosis in the mice fed a fast-food diet. (**A**) Hepatic hydroxyproline levels. (**B**) Sirius Red staining of the liver sections and quantification of the positively stained areas (%). Scale bar = 100 μm. (**C**) Immunohistochemical staining for α-smooth muscle actin (α-SMA) of the liver sections and quantification of the positively stained areas (%), as well as the hepatic transcript levels of collagen 1α1 (*Col1a1*), tissue inhibitor of metalloproteinase 1 (*Timp1*) and matrix metalloproteinase 2 (*Mmp2*). Scale bar = 100 μm. Tissue sections from all the mice were stained and quantified (means ± SEM). (**D**) Western blotting of α-SMA and transforming growth factor-β1 (TGF-β1) in the livers. Each four samples from the NCV-V/NCD-E groups and 9–10 samples from the FFD-V/FFD-E groups were used for Western blot quantification. NCD-V/NCD-E: The mice fed a normal chow diet (NCD) were injected with a vehicle (NCD-V, *n* = 6) or eritoran (NCD-E, *n* = 6); FFD-V/FFD-E: the mice fed a fast-food diet (FFD) were injected with a vehicle (FFD-V, *n* = 10) or eritoran (FFD-E, *n* = 9); ^#^ *p* < 0.05 vs. NCD-V; * *p* < 0.05 vs. FFD-V.

In addition, eritoran attenuated liver fibrosis induced by the FFD as determined based on the hepatic hydroxyproline levels (Figure 3A) and the positive Sirius Red-stained areas (Figure 3B). A significant reduction in protein levels of the marker of HSC activation, alpha smooth muscle actin (α-SMA), and the profibrotic marker TGF-β1 was observed in the livers of the FFD-E mice (Figure 3C,D). There were no significant changes in the hepatic transcript expression levels of collagen type 1α1, tissue inhibitor of metalloproteinase 1 (*Timp1*) and matrix metalloproteinase 2 (*Mmp2*) (Figure 3C). These findings suggested that eritoran attenuated FFD-induced liver inflammation and fibrosis with suppression of HSC activation.

### 3.2. Eritoran Did Not Reduce Hepatic Steatosis or Systemic Insulin Resistance in the FFD-Fed Mice

**Figure 4:**
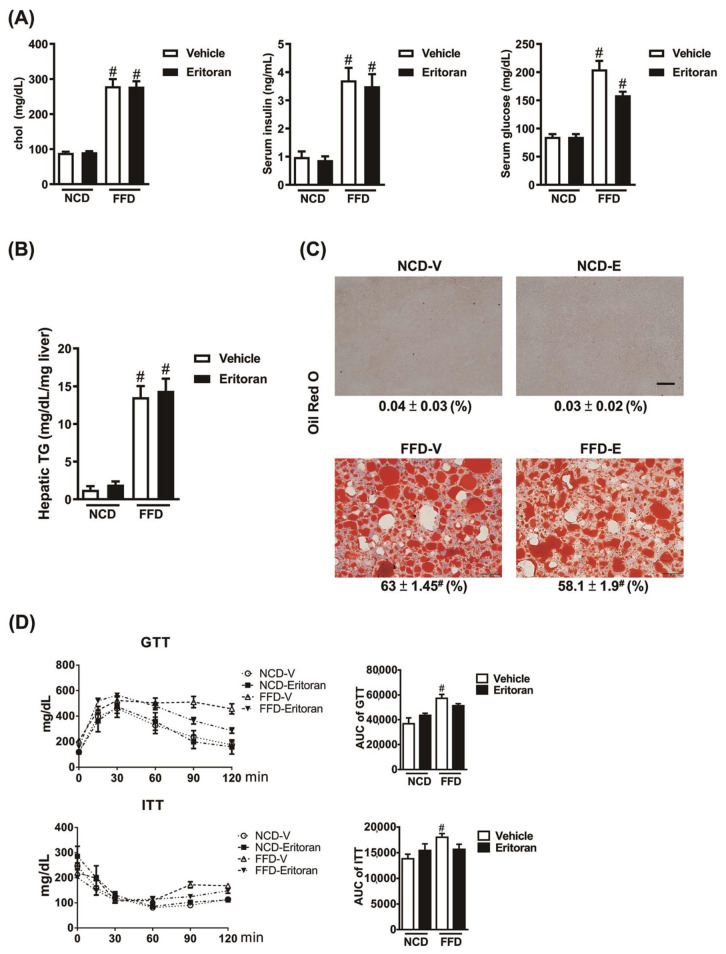
Eritoran did not reduce hepatic steatosis or systemic insulin resistance in the FFD-fed mice. (**A**) Serum cholesterol (chol), insulin and glucose levels. (**B**) Hepatic triglyceride (TG) levels. (**C**) Oil Red O staining of the liver sections and quantification of the stained areas (%). Scale bar = 50 μm. Tissue sections from all the mice were stained and quantified (means ± SEM). (**D**) Glucose tolerance testing (GTT) and insulin tolerance testing (ITT) of the groups. AUC: area under the curve. NCD-V/NCD-E: the mice fed a normal chow diet (NCD) were injected with a vehicle (NCD-V, *n* = 6) or eritoran (NCD-E, *n* = 6); FFD-V/FFD-E: the mice fed a fast-food diet (FFD) were injected with a vehicle (FFD-V, *n* = 10) or eritoran (FFD-E, *n* = 9); ^#^ *p* < 0.05 vs. NCD-V.

Serum cholesterol, insulin and glucose levels were significantly higher in the FFD-fed mice and were not decreased in the mice treated with eritoran (Figure 4A). Eritoran did not alter hepatic steatosis induced by the FFD (Figure 4B), which was further confirmed by the results obtained from H&E and Oil Red O staining of the liver sections (Figure 2E and Figure 4C). Glucose tolerance testing and insulin tolerance testing revealed that eritoran did not improve systemic insulin resistance (Figure 4D).

### 3.3. Hepatic TLR4 Signaling Pathway in the FFD-Fed Mice Was Suppressed by Eritoran

**Figure 5:**
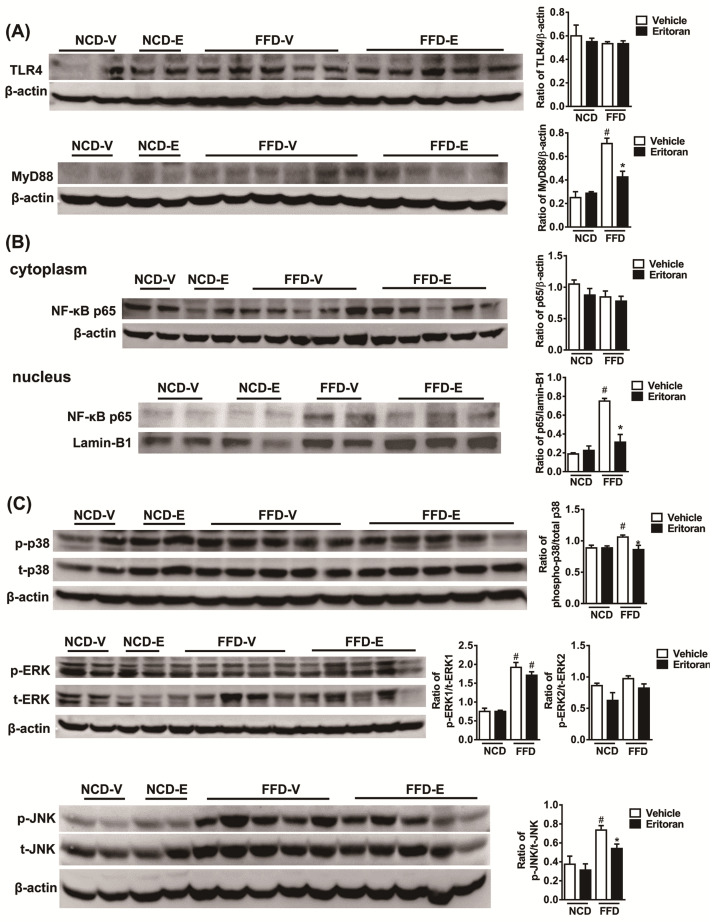
Eritoran suppressed the hepatic TLR4 downstream signaling pathway in the mice fed a fast-food diet. Western blot analysis and quantification of (**A**) TLR4 and myeloid differentiation factor 88 (MyD88); (**B**) cytoplasmic and nuclear NF-κB p65; (**C**) phosphorylated and total p38 (p/t-p38), extracellular signal-regulated kinase 1 and 2 (p/t-ERK1/2) and c-Jun N-terminal kinase (p/t-JNK) expression in the livers. NCD-V/NCD-E: the mice fed a normal chow diet (NCD) were injected with a vehicle (NCD-V, *n* = 6) or eritoran (NCD-E, *n* = 6); FFD-V/FFD-E: the mice fed a fast-food diet (FFD) were injected with a vehicle (FFD-V, *n* = 10) or eritoran (FFD-E, *n* = 9). Four samples from each of the NCV-V/NCD-E groups and 9–10 samples from the FFD-V/FFD-E groups were used for Western blot quantification; ^#^ *p* < 0.05 vs. NCD-V; * *p* < 0.05 vs. FFD-V.

To better define the mechanisms underlying the effect of eritoran, we next examined the downstream mediators of the TLR4 signaling pathway. Western blotting showed that eritoran decreased the hepatic MyD88 levels, although hepatic TLR4 expression was not reduced (Figure 5A). In addition, the nuclear translocation of NF-κB p65 was suppressed in the eritoran-treated FFD-fed mice (Figure 5B). The phosphorylation of p38 and c-Jun N-terminal kinase (JNK) was also reduced after eritoran treatment (Figure 5C). These results demonstrated that the chronic administration of eritoran successfully suppressed the hepatic TLR4 signaling pathway by downregulating the MyD88 expression, NF-κB p65 nuclear translocation and phosphorylation of p38 and JNK in a NASH mouse model.

### 3.4. Eritoran Attenuated Hepatic Inflammation and Fibrosis in the CCl_4_-Treated Mice

**Figure 6:**
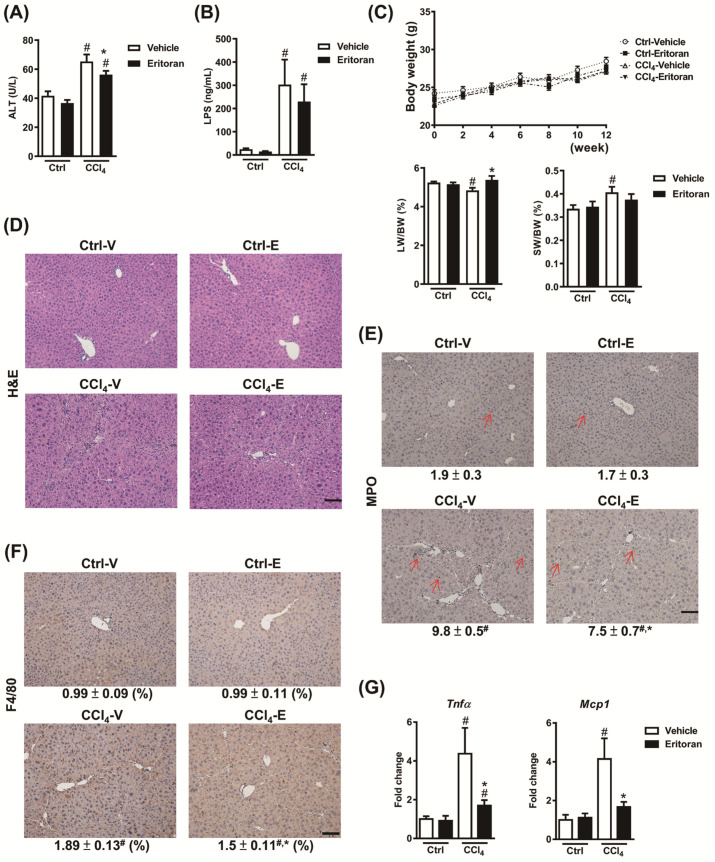
Eritoran decreased liver inflammation in the carbon tetrachloride (CCl_4_)-injured mice. (**A**) Serum ALT levels. (**B**) Serum LPS levels. (**C**) Liver weight (LW) and spleen weight (SW). (**D**) Hematoxylin and eosin (H&E), (**E**) myeloperoxidase (MPO) staining and (**F**) immunohistochemical staining for F4/80 of the liver sections and quantification of the MPO-positive stained cells (arrow) as well as the positively stained F4/80 areas (%). Scale bar = 100 μm. Tissue sections from all the mice were stained and quantified (means ± SEM). (**G**) Hepatic transcript levels of tumor necrosis factor-α (*Tnfa*) and monocyte chemoattractant protein 1 (*Mcp1*). Ctrl-V/Ctrl-E: the mice that received corn oil (Ctrl) were injected with a vehicle (Ctrl-V, *n* = 8) or eritoran (Ctrl-E, *n* = 8); CCl_4_-V/CCl_4_-E: the mice that received carbon tetrachloride (CCl_4_) were randomized to be injected with a vehicle (CCl_4_-V, *n* = 9) or eritoran (CCl_4_-E, *n* = 9); ^#^ *p* < 0.05 vs. Ctrl-V; * *p* < 0.05 vs. CCl_4_-V.

To validate the anti-inflammatory and antifibrotic effects of eritoran in chronic liver injury, we generated another liver fibrosis model using an intraperitoneal CCl_4_ injection. The CCl_4_-injured mice showed modestly elevated serum ALT levels, significantly increased serum LPS levels, decreased liver weight and increased hepatic neutrophil and macrophage infiltration (Figure 6A–F). Serum ALT levels were decreased by eritoran in the CCl_4_-injured mice, but the difference in LPS levels was not statistically significant (Figure 6A,B). The eritoran-treated CCl_4_ mice demonstrated a restored liver weight (Figure 6C) and a reduction in hepatic neutrophil and macrophage infiltration (Figure 6D–F). In addition, eritoran treatment significantly decreased the hepatic transcript levels of *Tnfa* and *Mcp1* (Figure 6G).

**Figure 7:**
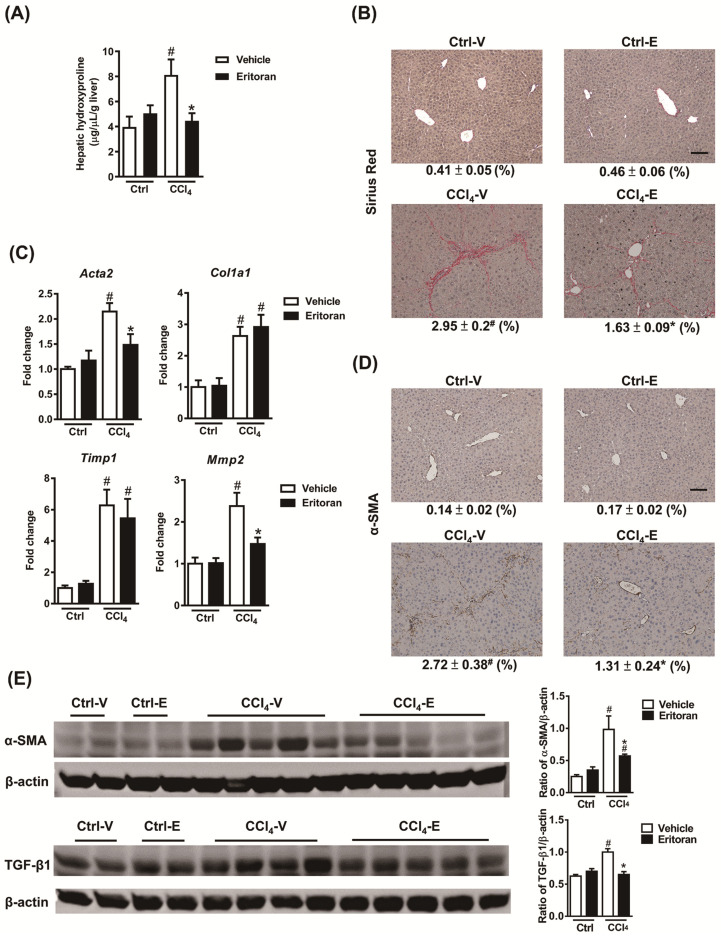
Eritoran attenuated liver fibrosis in the mice injured by CCl_4_. (**A**) Hepatic hydroxyproline levels. (**B**) Sirius Red staining of the liver sections and quantification of the positively stained areas (%). Scale bar = 100 μm. Tissue sections from all the mice were stained and quantified (means ± SEM). (**C**) Hepatic transcript levels of α-smooth muscle actin (*Acta2*), collagen 1α1 (*Col1a1*), tissue inhibitor of metalloproteinase 1 (*Timp1*) and matrix metalloproteinase 2 (*Mmp2*). (**D**) Immunohistochemical staining for α-SMA of the liver sections and quantification of the positively stained areas (%). Scale bar = 100 μm. Tissue sections from all the mice were stained and quantified (means ± SEM). (**E**) Western blotting of α-SMA and transforming growth factor-β1 (TGF-β1) in the livers. Ctrl-V/Ctrl-E: the mice that received corn oil (Ctrl) were treated with a vehicle (V) or eritoran (E); CCl_4_-V/CCl_4_-E: the CCl_4_-injured mice were treated with a vehicle (V) or eritoran (E); ^#^ *p* < 0.05 vs. Ctrl-V; * *p* < 0.05 vs. CCl_4_-V.

**Figure 8:**
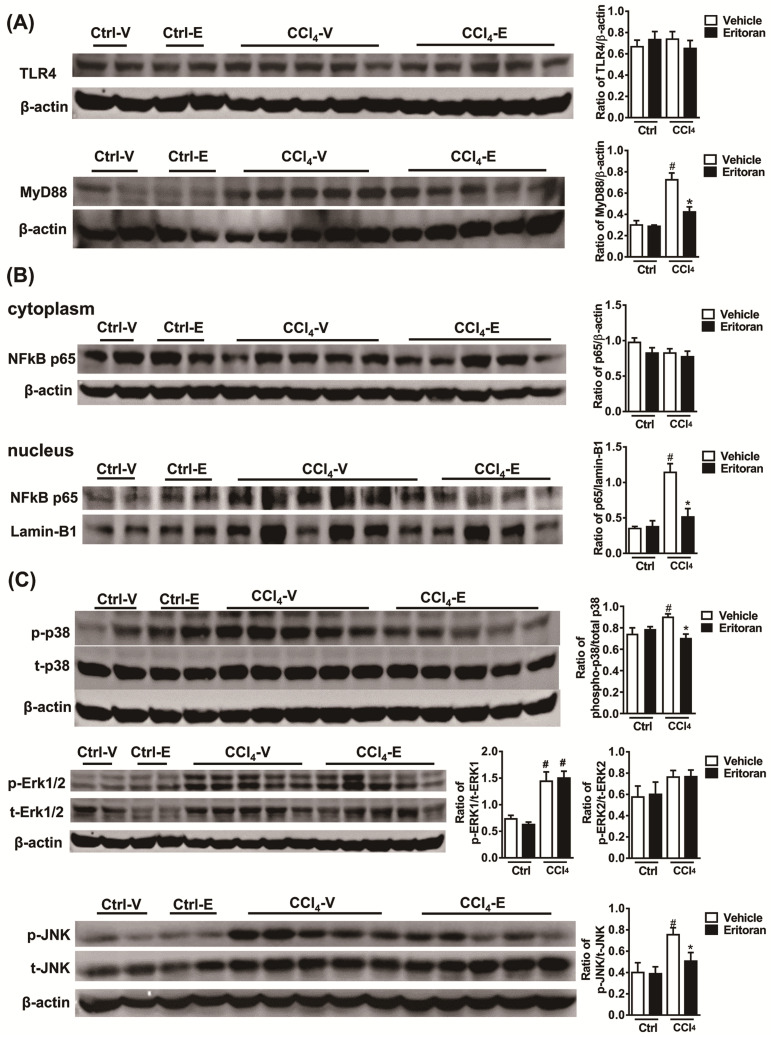
Eritoran suppressed the hepatic TLR4 signaling pathway in the mice treated with CCl_4_. Western blot analysis and quantification of (**A**) TLR4 and myeloid differentiation factor 88 (MyD88); (**B**) cytoplasmic and nuclear NF-κB p65; (**C**) phosphorylated and total p38 (p/t-p38), extracellular signal-regulated kinase 1 and 2 (p/t-ERK1/2) and c-Jun N-terminal kinase (p/t-JNK) expression in the livers. Ctrl-V/Ctrl-E: the mice that received corn oil (Ctrl) were treated with a vehicle (V) or eritoran (E); CCl_4_-V/CCl_4_-E: the CCl_4_-injured mice were treated with a vehicle (V) or eritoran (E). Four samples from each of the Ctrl-V/Ctrl-E groups and nine samples from the CCl_4_-V/CCl_4_-E groups were used for Western blot quantification; ^#^ *p* < 0.05 vs. Ctrl-V; * *p* < 0.05 vs. CCl_4_-V.

Notably, the hepatic hydroxyproline levels and Sirius Red staining results revealed that eritoran significantly ameliorated CCl_4_-induced liver fibrosis (Figure 7A,B). The hepatic expression levels of α-SMA and TGF-β1 were decreased in the eritoran-treated CCl_4_ mice (Figure 7C–E). Regarding hepatic TLR4 signaling, eritoran reduced the hepatic MyD88 expression (Figure 8A) and the nuclear translocation of NF-κB p65 (Figure 8B) in the CCl_4_-injured mice. The phosphorylation of p38 and JNK was also reduced by eritoran without altering the phosphorylation of ERK (Figure 8C). These findings were consistent with the findings in the FFD experiment and indicated that eritoran suppressed the hepatic TLR4 signaling pathway in animal models of chronic liver injury.

### 3.5. Eritoran Suppressed the NF-κB p65 Nuclear Translocation in HSCs and KCs

**Figure 9:**
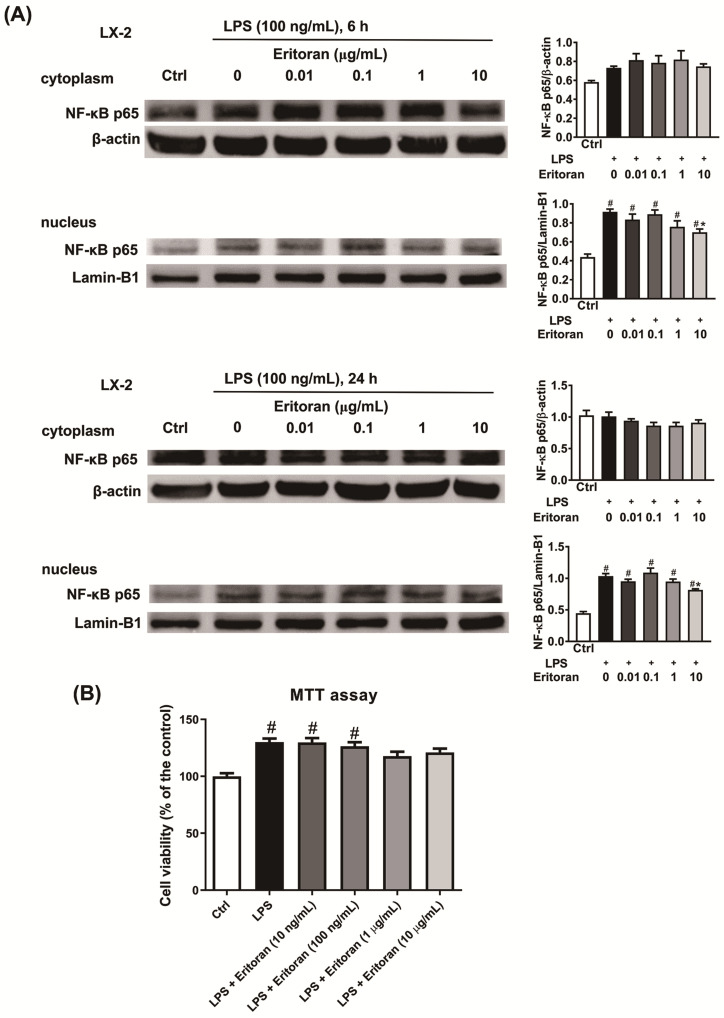
The NF-κB p65 nuclear translocation in LX-2 cells. (**A**) Incubation of LX-2 cells (2 × 10^6^/well) with lipopolysaccharide (LPS, 100 ng/mL) and with a serial dose of eritoran (0–10 μg/mL) for 6 h and 24 h. Western blotting of nuclear and cytoplasmic protein of NF-κB p65 was performed (*n* = 4/group). (**B**) Cell viability determined by the methyl thiazolyl tetrazolium (MTT) assay in LX-2 cells (1 × 10^4^/well) incubated with the control medium (Ctrl), LPS (100 ng/mL) and a serial concentration of eritoran (0–10 μg/mL) for 24 h (*n* = 8/group); ^#^ *p* < 0.05 vs. the Ctrl group; * *p* < 0.05 vs. the group treated with LPS and without eritoran.

First, the ability of eritoran to block the LPS-induced NF-κB p65 nuclear translocation in an activated human HSC cell line, LX-2 cells, was tested. Incubation of LX-2 cells with LPS for 6 and 24 h resulted in an increase in the NF-kB p65 nuclear translocation (Figure 9A), which was markedly downregulated by coincubation with eritoran at the dose of 10 μg/mL. In the MTT assay, the addition of 10 ng/mL eritoran to 10 μg/mL did not significantly decrease the viability of the LX-2 cells coincubated with LPS (Figure 9B). Therefore, eritoran at the dose of 10 μg/mL was used in the primary liver cell experiments.

**Figure 10:**
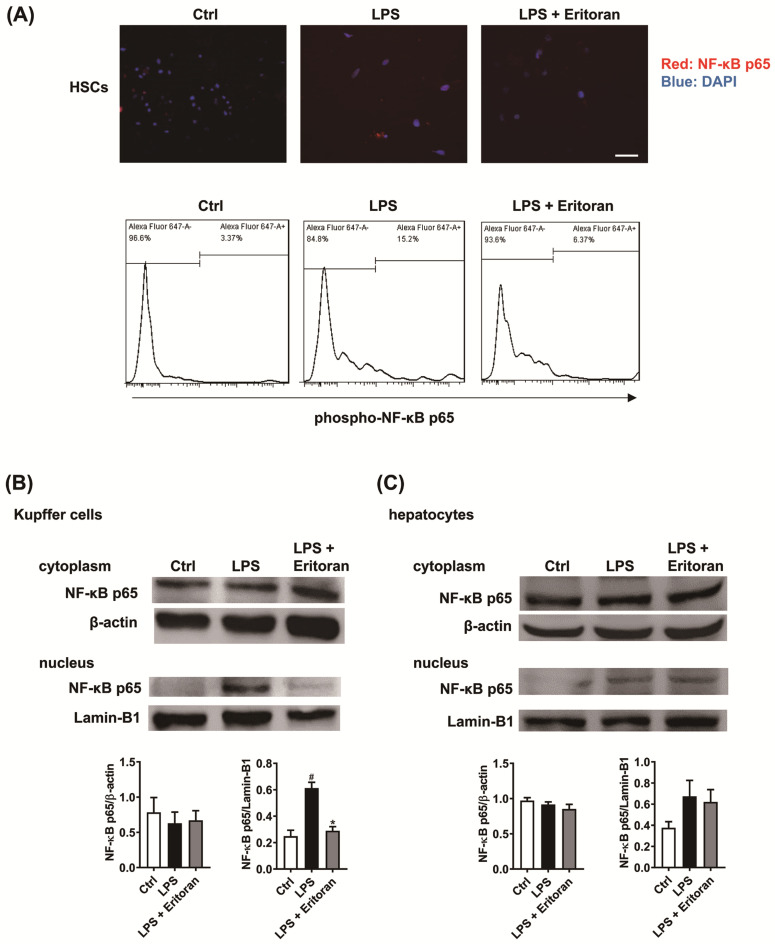
Eritoran suppressed the LPS-induced nuclear translocation of NF-κB p65 in primary hepatic stellate cells (HSCs) and Kupffer cells (KCs). (**A**) Immunofluorescence staining of primary mouse HSCs treated with the control medium (Ctrl), lipopolysaccharide (LPS, 100 ng/mL) or LPS (100 ng/mL) + eritoran (10 μg/mL) for 24 h. NF-κB p65, red; DAPI (diamidino-2-phenylindole) nuclear stain, blue. Scale bar = 50 μm. Flow cytometric analysis of the phosphorylated NF-κB p65 expression in primary HSCs. (**B**) Cytoplasmic and nuclear NF-κB p65 of primary KCs treated with the Ctrl, LPS (100 ng/mL) or LPS (100 ng/mL) + eritoran (10 μg/mL) for 24 h (*n* = 4/group). (**C**) Cytoplasmic and nuclear NF-κB p65 of primary hepatocytes treated with the Ctrl, LPS (1 μg/mL) or LPS (1 μg/mL) + eritoran (10 μg/mL) for 24 h (*n* = 4/group); ^#^ *p* < 0.05 vs. the Ctrl group; * *p* < 0.05 vs. the LPS group.

Immunofluorescence and flow cytometry showed that eritoran decreased the LPS-induced NF-κB p65 nuclear translocation in primary mouse HSCs (Figure 10A). Western blotting of primary KCs and hepatocytes revealed that the LPS-induced NF-κB p65 nuclear translocation in KCs was significantly suppressed by eritoran (Figure 10B) but was not altered by eritoran in hepatocytes (Figure 10C). These findings indicate that eritoran is effective in diminishing the TLR4-mediated response to LPS in primary HSCs and KCs.

### 3.6. Eritoran Decreased MyD88-Mediated p38/JNK Phosphorylation in Kupffer Cells

**Figure 11:**
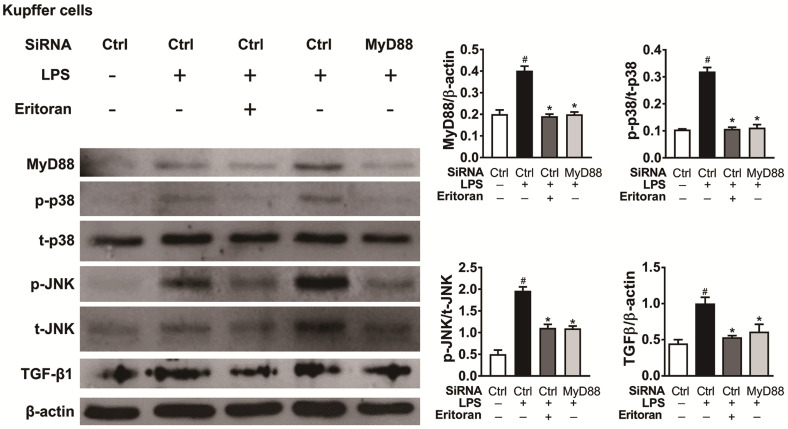
Eritoran decreased the MyD88-mediated p38/JNK phosphorylation in KC cells. Western blot analysis and quantification of myeloid differentiation factor 88 (MyD88), phosphorylated and total p38 (p/t-p38) and c-Jun N-terminal kinase (p/t-JNK) and transforming growth factor-β1 (TGF-β1) of primary KCs from the different treatment (*n* = 4/group). The isolated KCs were incubated with the siRNA control (Ctrl) (30 nM) or MyD88-siRNA (30 nM) for 48 h. After transfection, the cells were treated with or without LPS (10 ng/mL) or eritoran (10 μg/mL) for 6 h; ^#^ *p* < 0.05 vs. the group treated only with the siRNA control; * *p* < 0.05 vs. the group treated with the siRNA control and eritoran.

Incubation of isolated KCs with LPS for 6 h increased the expression of MyD88 and the phosphorylation of p38 and JNK downregulated by coincubation with eritoran or treatment of MyD88-siRNA. In addition, the LPS-induced increased expression of TGF-β1 in KCs was decreased by the eritoran or MyD88-siRNA treatment (Figure 11). These findings implicate that eritoran is effective in suppressing TLR4 signaling by reducing the response of the MyD88-mediated p38/JNK phosphorylation to LPS in primary KCs.

## 4. Discussion

This is the first study to demonstrate that chronic administration of eritoran exerted anti-inflammatory and antifibrotic effects in the mice with chronic liver injury. In the NASH model, our findings indicated that eritoran attenuated liver inflammation by improving the hepatic neutrophil accumulation and the proinflammatory cytokine and chemokine expression without altering hepatic steatosis and insulin resistance. In addition, eritoran ameliorated liver fibrosis through suppression of HSC activation with decreased expression of profibrotic markers α-SMA and TGF-β1. The effects of eritoran were mediated by the successful suppression of the hepatic TLR4 downstream pathway and blocking of LPS-TLR4 signaling in HSCs and KCs and were examined in two liver fibrosis mouse models. Inhibition of TLR4 has been suggested to ameliorate hepatic injury and progression to NASH in animal studies [29,30]. However, JKB-121, a weak antagonist of TLR4, recently failed to show a beneficial effect on histology in patients with NASH in a small phase II randomized trial [31]. Our findings suggested that eritoran may be a promising alternative candidate to use in patients with liver fibrosis in clinical trials.

TLR4 is an attractive therapeutic target of CLD as it is involved in several hepatic inflammatory and fibrotic processes induced by various etiologies, such as NASH and alcoholic liver disease, as well as in animal models of chronic liver injury [29,30,32,33]. Pharmacological inhibition of TLR4 with eritoran or other inhibitors has been investigated in several acute liver injury models, including ischemia-reperfusion [20], trauma/shock [21] and acute-on-chronic liver failure models [34], which showed that TLR4 antagonists could suppress TLR4 signaling, the subsequent inflammatory cascade and liver injury in vivo. The translation of eritoran into humans with sepsis reached phase III trials [35], which confirmed the safety of the drug, although eritoran failed to show a significant difference in the 28-day survival. We first demonstrated that chronic administration of eritoran not only ameliorated liver inflammation, which is consistent with the findings obtained in acute liver injury models [20,21], but also attenuated liver fibrosis in NASH and CCl_4_ models. Liver fibrosis is the most important prognostic indicator in patients with nonalcoholic fatty liver disease [36,37] or other CLD [38]. Therefore, our findings suggest that eritoran may serve as a potential drug for CLD.

**Figure 12:**
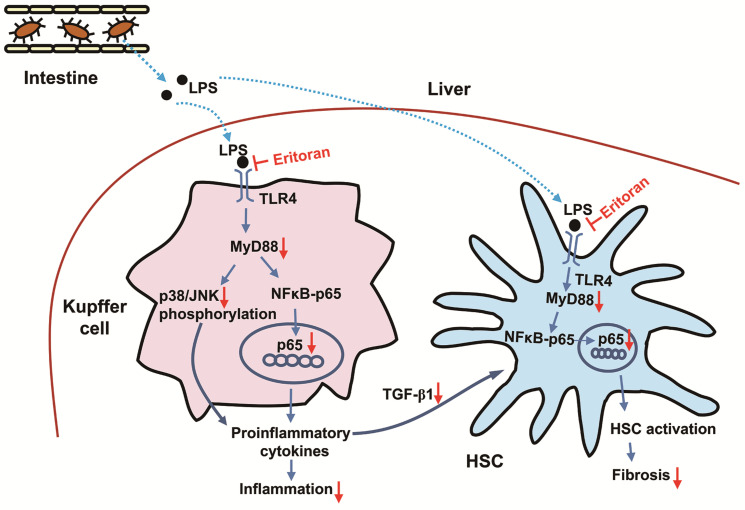
The proposed scheme of the regulation mechanism of eritoran in mice with chronic liver injury. The increased lipopolysaccharide (LPS) due to the increased gut permeability in the fast-food diet-fed or tetrachloride-injured mice [45,46] entered the liver and bound toll-like receptor 4 (TLR4) on the Kupffer cells or hepatic stellate cells (HSCs). In Kupffer cells, LPS activated myeloid differentiation factor 88 (MyD88), leading to the NF-κB p65 nuclear translocation and increased phosphorylation of p38 and c-Jun N-terminal kinase (JNK), which contributed to increased production of proinflammatory cytokines and inflammation. The secreted transforming growth factor-β1 (TGF-β1) from Kupffer cells could promote HSC activation. On the other hand, LPS binding to TLR4 of HSCs could also activate MyD88, leading to the NF-κB p65 nuclear translocation, which contributed to HSC activation and liver fibrosis [10,11,12,39]. Eritoran blocked the TLR4 signaling pathway by competing with LPS for binding sites on Kupffer cells and HSCs, leading to downregulation of the MyD88-dependent NF-κB and JNK/p38 pathways, which contributed to attenuation in hepatic inflammation and fibrosis. Blue arrow: upregulation; red arrow: downregulation.

TLR4 signaling in KCs induces proinflammatory and profibrogenic cytokine production [10] through downstream pathways, including the activation of NF-κB [39], and contributes to liver inflammation in NASH [6] as well as other CLD [12]. In addition, LPS-TLR4 activation in HSCs is considered essential for hepatic fibrogenesis [12]. Activated human HSCs respond to LPS via the activation of NF-κB and JNK and the secretion of proinflammatory cytokines [40]. In murine quiescent HSCs, TLR4 activation not only upregulates chemokine secretion and induces the chemotaxis of KCs, but also downregulates the TGF-β pseudoreceptor Bambi to sensitize HSCs to TGF-β-induced signals stimulating collagen production, which is mediated via a MyD88–NF-κB-dependent pathway [11]. Previous studies reported that eritoran reduced the production of TNF-α in the macrophage cell line RAW 264.7 [20]. The effect of eritoran on HSCs had not been previously investigated. In our study, eritoran successfully suppressed the LPS-induced nuclear translocation of NF-κB in a human activated HSCs cell line, LX-2 cells, primary HSCs and KCs. The findings suggested that the anti-inflammatory and antifibrotic effects of eritoran in chronic liver injury models resulted from the suppression of TLR4 signaling in KCs and HSCs (Figure 12).

Shi et al. reported that mice lacking TLR4 were partially protected from insulin resistance induced by lipid infusion and a high-fat diet (HFD) [41,42]. TLR4 deficiency also protected mice from methionine choline-deficient diet-induced liver fat deposition [43] and hepatocyte-specific TLR4 knockout ameliorated hepatic steatosis in the HFD-fed mice [42]. A recent in vitro study showed that the LPS-induced NF-κB p65 nuclear translocation in a human hepatocyte cell line, HepaRG cells, was partially attenuated by a TLR4 inhibitor, CLI-095 [44]. However, in our study, the pharmacological inhibition of TLR4 using eritoran did not improve systemic insulin resistance and hepatic steatosis in the FFD-fed mice. In our in vitro study, the LPS-induced nuclear translocation of NF-κB p65 in primary mouse hepatocytes was not reversed by eritoran either. The discrepant findings from the in vitro studies may result from the different cells and drugs used. Our findings indicate that the anti-inflammatory and antifibrotic effects of eritoran in the NASH model was not attributed to the improvement of hepatic steatosis or insulin resistance, and there may be different sensitivities to eritoran in terms of NF-κB activation in liver parenchymal and nonparenchymal cells.

## 5. Conclusions

In conclusion, this study demonstrated that chronic administration of eritoran significantly attenuated liver inflammation and fibrosis in the FFD- and CCl_4_-indued liver fibrosis models by suppressing the MyD88–NF-κB pathway in the liver, HSCs and KCs. Therefore, targeting TLR4 with eritoran could be a potential treatment strategy for patients with CLD.

## Data Availability

All the data generated and analyzed during this study are included in this article and its Supplementary Information files.

## Supplementary Materials

The following are available online at https://www.mdpi.com/article/10.3390/cells10061562/s1, Table S1: Primers used for quantitative RT-PCR, Table S2: Antibody details and conditions used for Western blotting and immunostaining.
