# Supplementary material for: Eritoran Attenuates Hepatic Inflammation and Fibrosis in Mice with Chronic Liver Injury
Source: Cells. 2021 Jun 21;10(6):1562. doi: 10.3390/cells10061562 (PMC8235164; doi:10.3390/cells10061562)
Supplement: Supplementary file 1 [file cells-10-01562-s001.zip › cells-1216210-supplementary.pdf]

Table S1. Primers used for quantitative RT-PCR.

Gene name	Primer	Sequence	Size (bp)
Mouse			
Mcp1	Forward	5′- ACCACAGTCCATGCCATCAC-3′	986
	Reverse	5′- TTGAGGTGGTTGTGGAAAAG-3′	
Tgfb1	Forward	5′- GTGGAAATCAACGGGATCAG-3′	229
	Reverse	5′- ACTTCCAACCCAGGTCCTTC-3′	
Mmp2	Forward	5′- CTGATAACCTGGATGCCGTCGT-3′	117
	Reverse	5′- TGCTTCCAAACTTCACGCTCTT-3′	
Acta2	Forward	5′- GTCCCAGACATCAGGGAGTAA-3′	102
	Reverse	5′- TCGGATACTTCAGCGTCAGGA-3′	
Col1a1	Forward	5′- GAGCGGAGAGTACTGGATCG-3′	158
	Reverse	5′- GCTTCTTTTCCTTGGGGTTC-3′	
Timp1	Forward	5′- CCAGAACCGCAGTGAAGAG-3′	183
	Reverse	5′- CAA GGG ATA GAT AAA CAG GGA-3′	
Tnfa	Forward	5′- TGCCTATGTCTCAGCCTCTTC-3′	117
	Reverse	5′- GAGGCCATTTGGGAACTTCT-3′	
Gapdh	Forward	5′- TGTTGAAGTCGCAGGAGACAACCT-3′	111
	Reverse	5′- AACCTGCCAAGTATGATGACATCA-3′	

Table S2. Antibody details and conditions used for western blotting and immunostaining

Antibody	Supplier	Catalog no.	Application	Dilution
β-actin	GeneTex	GTX629630	WB	1:10000
Lamin B1	Cell Signaling	#12586	WB	1:1000
α-SMA	GeneTex	GTX100034	WB	1:1000
			IHC	1:700
p-ERK1/2	Cell Signaling	#9101	WB	1:1000
t-ERK1/2	Cell Signaling	#9102	WB	1:1000
p-p38	Cell Signaling	#9216	WB	1:1000
t-p38	Cell Signaling	#9212	WB	1:1000
p-JNK	Cell Signaling	#4668	WB	1:1000
t-JNK	Cell Signaling	#9252	WB	1:1000
TLR4	GeneTex	GTX13556	WB	1:1000
MyD88	GeneTex	GTX112987	WB	1:5000
NF-κB p65	GeneTex	GTX102090	WB	1:1500
			IF	1:200
NF-κB pp65	Cell Signaling	#3033	FCM	1:1600
TGF-β1	Abcam	ab92486	WB	1:1000
F4/80	Abcam	ab6640	IHC	1:400
MPO	Abcam	ab9535	IHC	1:50

Antibodies were purchased from Cell Signaling (Beverley, MA, USA), Abcam (Cambridge, MA, USA), GeneTex (Irvine, CA, USA). WB: western blotting; IHC: immunohistochemistry; IF: immunofluorescence; FCM: flow cytometry
